# Quantitative ultrasound applied to metacarpal bone in infants

**DOI:** 10.7717/peerj.141

**Published:** 2013-08-27

**Authors:** Francesco Savino, Serena Viola, Stefania Benetti, Simone Ceratto, Valentina Tarasco, Maria Maddalena Lupica, Luca Cordero di Montezemolo

**Affiliations:** Azienda Ospedaliera Città della Salute e della Scienza di Torino, Ospedale Infantile Regina Margherita, Torino, Italy; Dipartimento di Scienze della Sanità Pubblica e Pediatriche, Università degli Studi di Torino, Italy

**Keywords:** Quantitative ultrasound, Second metacarpus, Reference values, Bone mineral status, Rickets, Infants

## Abstract

**Aim.** To provide bone status assessment in infancy using quantitative ultrasound (QUS) applied to second metacarpus.

**Methods.** 103 healthy term infants and 3 patients with rickets, aged ≤ 12 months, underwent metacarpal QUS evaluation using QUS DBM Sonic Aurora IGEA (MO, Italy), which measures speed of sound (mcSOS) and bone transmission time (mcBTT).

**Results.** In the total sample, median (interquartile range) of mcSOS was 1640.00 (26.0) m/s and mcBTT 0.82 (0.21) µs. Moreover, reference values for age were obtained based on estimation of the lower and upper percentiles. We observed a statistical significant difference between groups of age for mcSOS (*p* = 0.016). In a multiple linear regression model, we found a relation between age at enrolment and mcSOS (β = −0.608; *p* = 0.000) and mcBTT (β = −0.819; *p* = 0.001). A positive correlation between mcSOS and mcBTT has been observed (*r* = 0.631; *p* = 0.000). All the patients with rickets showed values of mcSOS and mcBTT lower than the 10th percentile.

**Conclusion.** Our findings show that this new simple technique appears to be a promising tool for monitoring bone mineral status in pediatric clinical practice and in early life. Furthermore, it could be considered a useful method to investigate and to monitor the role of different factors on programming of bone health and it should be tested as a new method for monitoring subjects with rickets during therapy.

## Introduction

The measurement of bone mineral status in early life may be a useful tool in identifying metabolic, genetic, and environmental factors important to determine whether or not children acquire an appropriate amount of bone for their body size. Optimizing bone mineral accrual may prevent poor mineral accretion and childhood fractures, and possibly delay the development of osteoporosis later in life (McDevitt & Ahmed, 2007).

Several methods are available for assessing bone mineralization. Presently, dual energy X-ray absorptiometry (DXA) is the most extensively used technique because it provides a good surrogate measure of bone health (Zemel et al., 2011). However, the exposure to ionizing radiation is an important limiting factor for preventive studies in large populations of children. Further limitations are lack of standardization of measurements for paediatric age (Picaud et al., 2003), and mismatch between mineral accretion and bone growth (Rauch & Schoenau, 2002).

Ultrasound may be particularly indicated to assess bone mineral status in children and neonates. Quantitative ultrasound (QUS) techniques are non-invasive, safe, radiation-free and easy to use (Guglielmi, Adams & Link, 2009). In recent years, studies demonstrated that the radius, humerus and tibia can be used as sites of measurement in preterm and term infants (Baroncelli et al., 2003; Nemet et al., 2001; Rubinacci et al., 2003; Gonnelli et al., 2004). More recently, QUS has been also applied at the midshaft of the second metacarpal bone in newborns. The following advantages have been observed: easy access (infant hands fit with the flat surfaces of the transducers), compliance and safety because the infant can withdraw the hand without risk of injury, suitable also for preterm infants due to the very early ossification of the metacarpal bone during foetal development (Ritschl et al., 2005).

Therefore, in infancy, a non-irradiating method which takes into account not only bone density, but also bone mechanical properties and microstructure, geometry and porosity of cortical bone would be desirable. Thus, QUS could be an important method to evaluate bone mineral status in infants with disorders of bone metabolism, such as rickets, that is a disease of deficient mineralization of cartilage and osteoid, and is also characterized by retarded endochondral ossification, which causes excessive accumulation of physeal cartilage, growth failure and skeletal deformities (Mughal, 2011).

The aim of the present study was to provide bone status assessment in infancy using QUS applied to the second metacarpus.

## Patients and Methods

### Study design and subjects

103 healthy infants (53 males and 50 females) were consecutively recruited at the Department of Pediatrics, Regina Margherita Children Hospital – Azienda Ospedaliera Città della Salute e della Scienza di Torino. We included Caucasian subjects with apparently normal skeletal status, aged ≤ 12 months, born at term with birth weight appropriate for gestational age (2500–4000 g) and Apgar score ≥ 7 at five minutes. The following exclusion criteria were considered: history of congenital or perinatal disorders, intrauterine growth retardation, congenital infection syndromes, acute or chronic growth diseases, bone pathologies, signs or symptoms of dehydration, intake of steroidal (in particular, dexamethasone) or anticonvulsive drugs, maldigestion and malabsorption, neuromuscular, renal or endocrinological pathologies.

Furthermore, we performed QUS assessment in a small group of patients with rickets (*n* = 3) observed in our department during the study period.

After receiving informed consent from the parents and checking clinical medical history, all infants were examined by a paediatrician. Then, QUS assessment was performed with DBM Sonic Aurora (IGEA, Italy) by an experienced and specially trained operator on the left hand of the infants.

Infants were fed with breast-milk enriched with 400 IU of vitamin D or with formula; infants aged > 6 months were weaned following WHO/UNICEF Guidelines on Complementary Feeding (Brown, 2000).

The study was approved by the Local Ethics Committee prior to the start of the study and written informed consent was obtained from parents.

### Anthropometric parameters

All infants were weighted naked, before feeding, by means of an electronic integrating scale (SECA model 757, Vogel & Halke, Hamburg, Germany; precision: ± 1.0 g). Crown-to-heel length was measured using a Harpenden stadiometer (Holtain, Crymych, UK) and body mass index (BMI) was calculated (weight in kilograms divided by height in square meters [kg/m^2^]).

### QUS assessment

The subjects underwent QUS evaluation with the device for metacarpal quantitative ultrasonography DBM Sonic Aurora (IGEA, Italy). The device has an emitting transducer that generates 1.25-MHz pulses and a receiving transducer. The acoustic output energy is 1.8 mW/cm^2^. Warm gel was applied on the surface of each transducer that was in contact with the region of interest for each series of measurements.

**Figure 1:**
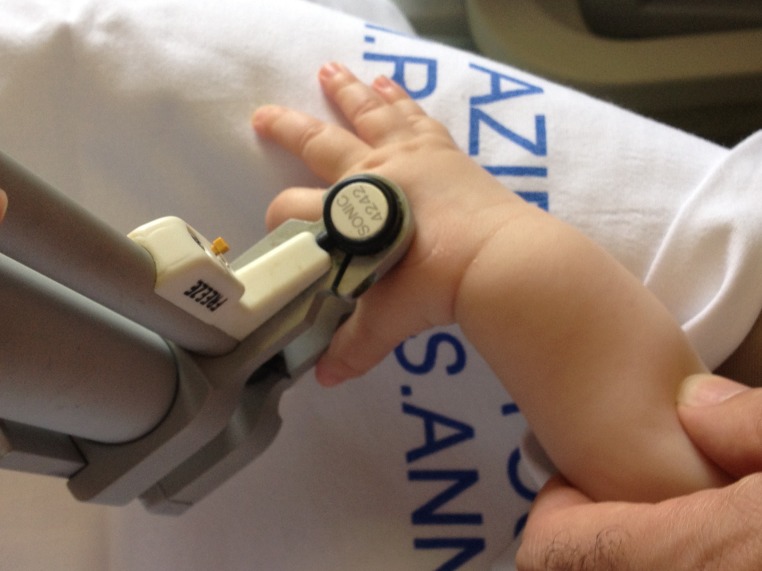
Application procedure.

The device can measure the following ultrasound parameters: “speed of sound” (SOS) and “bone transmission time” (BTT). SOS (m/s) is the velocity of ultrasound wave propagation between transmitting and receiving transducers. The sensitivities of the measurements are 0.01 mm and 0.05 µs, respectively. Ultrasound velocity depends on bone tissue density, elasticity, and structure. BTT (µs) attempts to minimise the confounding effect of soft tissue and is calculated as the difference between the arrival time at the receiving probe of the fastest ultrasound pulse, traveling through bone tissue, and the arrival time of the ultrasound pulse traveling through soft tissue only (Ritschl et al., 2005; Baroncelli, 2008). In our study, the selected site of measurement was the midshaft of the second metacarpal bone, thus, the parameters are specified respectively as mcSOS and mcBTT. Probes were positioned dorsal and volar on the hand up on the shaft of the second metacarpal bone (Fig. 1).

Four consecutive acquisitions were performed for each infant and the final value is the average of the four mcSOS measurements. The four mcSOS values had to lie within a range ≤ 10 m/s. During the measurements, the device displays the results of the mcSOS. If one acquisition was > 10 m/s, this measurement had to be repeated: if the repeated acquisition was not within the range, the measurement was discarded.

### Statistical analysis

All statistical analysis were performed using commercially available software SPSS 17.0 for Windows (SPSS Inc.; Chicago, IL, USA). Both anthropometric and QUS data are expressed as median and interquartile range (IR). To build up reference values, median and 10th–90th percentile were calculated.

Differences between groups in baseline characteristics and QUS parameters were determined using Mann–Whitney test or Kruskal Wallis as appropriate. Study correlations were investigated by Spearman’s correlation. Multiple linear regression was used to evaluate the relationship between QUS parameters and anthropometric variables to consider potentially confounding factors.

Level of statistical significance was set at *P* ≤ 0.05.

## Results

103 healthy infants were evaluated. The age of the study participants was 127 (181) days. Both genders were equally represented (53 males and 50 females), and the median age did not differ significantly between males and females.

At birth, weight was 3250 (570) g, length was 49.0 (2.0) cm, head circumference was 34.0 (2.0) cm and BMI was 13.3 (1.30) kg/cm^2^.

At enrolment, the anthropometric parameters were: weight 6540 (2983) g, length 62.0 (13.0) cm and BMI 15.3 (3.30) kg/cm^2^.

First, we performed the QUS measurements on the whole sample of infants. The measured values were mcSOS 1640.0 (26.0) m/s and mcBTT 0.82 (0.21) µs. Therefore, the relationships between QUS parameters and gender, birth season and age, weight, length and BMI at enrolment were evaluated.

The ultrasound parameters divided on the basis of gender (male *vs* female) were consecutively reported: mcSOS 1642.0 (27.5) m/s *vs* 1637.5 (22.5) m/s and mcBTT 0.85 (0.21) µs *vs* 0.76 (0.22) µs. QUS parameters did not significantly differ for gender.

Then, we evaluated mcSOS and mcBTT values distinguishing on the basis of different birth seasons. We found the following results:

– Spring (*n* = 33): mcSOS 1638.0 (19.0) m/s, mcBTT 0.83 (0.19) µs;
– Summer (*n* = 25): mcSOS 1637.0 (49.5) m/s, mcBTT 0.78 (0.37) µs;
– Autumn (*n* = 15): mcSOS 1640.0 (25.0) m/s, mcBTT 0.88 (0.16) µs;
– Winter (*n* = 30): mcSOS 1640.0 (32.0) m/s, mcBTT 0.85 (0.14) µs.

QUS parameters did not significantly differ for birth season.

**Figure 2:**
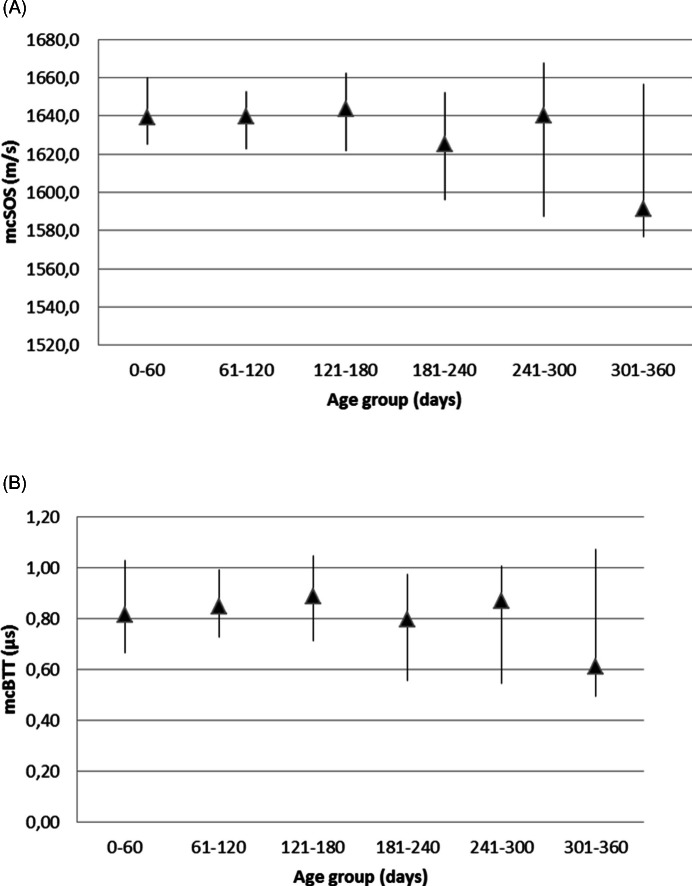
Reference values for mcSOS (A) and mcBTT (B) parameters, according to age.

We evaluated QUS parameters in different age groups as they are indexes of skeletal maturation. We observed a statistical significant difference between these groups for mcSOS parameter (*p* = 0.007) and for mcBTT (*p* = 0.05). We obtained reference values for mcSOS and mcBTT parameters, according to age, based on estimation of the lower and upper percentiles (Table 1, Figs. 2A and 2B).

**Table 1:** QUS parameters in different age groups.

*n*	Age (days)	Number of patients	mcSOS (m/s)	mcBTT (µs)		
			Median (IR)	10th–90th percentile	Median (IR)	10th–90th percentile
1	0–60	22	1639.0(23.25)	(1625.3; 1660.0)	0.82(0.27)	(0.67; 1.03)
2	61–120	24	1639.5(19.75)	(1622.5; 1652.5)	0.85(0.13)	(0.73; 0.99)
3	121–180	16	1643.5(15.50)	(1621.6; 1662.3)	0.89(0.21)	(0.71; 1.05)
4	181–240	12	1625.0(45.75)	(1595.9; 1652.4)	0.80(0.07)	(0.56; 0.97)
5	241–300	16	1640.0(35.00)	(1587.4; 1667.8)	0.87(0.25)	(0.54; 1.01)
6	301–360	13	1591.0(65.00)	(1576.8; 1656.8)	0.61(0.36)	(0.50; 1.07)

**Table 2:** QUS parameters in infants with rickets.

Patient	Age (days)	mcSOS (m/s)		mcBTT (µs)	
		Value	10th–90th percentile (age related)	Value	10th–90th percentile (age related)
1	370	1543	(1576.8; 1656.8)	0.41	(0.50; 1.07)
2	361	1570	(1576.8; 1656.8)	0.39	(0.50; 1.07)
3	232	1568	(1595.9; 1652.4)	0.36	(0.56; 0.97)

In the total sample, correlations with age were *r* = −0.350 (*p* < 0.001) for mcSOS and *r* = −0.296 (*p* = 0.002) for mcBTT, correlations with length were *r* = −0.268 (*p* = 0.006) for mcSOS and *r* = −0.198 (*p* = 0.04) for mcBTT and correlations with weight was *r* = −0.269 (*p* = 0.006) for mcSOS.

In a multiple linear regression model of QUS parameters between age, weight, length and BMI at enrolment, a relationship was found between age and mcSOS (β = −0.608; *p* < 0.001) and mcBTT (β = −0.819; *p* = 0.001).

A positive correlation between mcSOS and mcBTT has been observed (*r* = 0.631; *p* < 0.001).

All the patients with rickets showed values of mcSOS and mcBTT lower than 10th percentiles; mcSOS median (IR) was 1568 (27) and mcBTT median (IR) was 0.39 (0.05) (Table 2).

## Discussion

In literature, few studies have investigated bone health during the first months of life, considering the risk related to the most used technique in this field (DXA) that implies the exposure to ionizing radiation. QUS methods allow the study of skeletal development in early life in a safe and less invasive way (McDevitt & Ahmed, 2007).

Moreover, QUS measures some structural and qualitative parameters of bone, like cortical porosity, and trabecular connectivity to estimate bone mineral density while DEXA measures calcium content per unit of area (Camozzi et al., 2003; Njeh et al., 2001).

Our study provided an assessment of bone status in infants aged ≤ 12 months through QUS technique applied at the second metacarpal bone. Previously only one study has been performed using this site of application in healthy term infants (Ritschl et al., 2005), therefore no reference parameters are available. Another recent study used this technique but in preterm infants with parenteral aminoacid intakes (Scattolin et al., 2013). We have recently reported data about the influence of vitamin D supplementation on bone mineralization in exclusively breast-fed infants using this method (Savino et al., 2011).

We performed the analysis on the whole sample of infants.

Firstly, gender-related values were analyzed. No statistically significance in differences between QUS parameters in either gender was found. Similar results have been found in the study performed by Baroncelli et al. in children aged 2–8 years, suggesting that gender differences in bone mineralization could emerge later in life, in particular at pubertal age (Baroncelli et al., 2006). These findings have been recently confirmed by Lavado-Garcia et al. that observed a significant increase in ultrasound parameters with age (Lavado-Garcia et al., 2012).

Different factors have been found to have a significant impact on bone composition; among these, seasonal differences in bone mineral indices have been not much studied in infants. In children and possibly in adults, vitamin D metabolism presents seasonal variations. A recent study showed a seasonal variation in serum 25-hydroxyvitamin D (25(OH)D) levels of infants, despite supplementation with 400 IU of vitamin D daily (Halicioglu et al., 2012).

In our study we found no statistically significant differences in ultrasound parameters between children born in different seasons, although children born in spring and summer showed lower values than those born in autumn and winter. A study performed by Namgung et al. (1998) reported marked seasonal differences in newborn mineral content; in particular, it has been observed that summer-born infants had significantly lower values than winter-born ones. More recently, Liao et al. (2005) confirmed this seasonal trend, even though with a difference only in the order of 2%. These findings support the hypothesis that seasonal changes in serum concentrations of hormones regulating the metabolism of calcium and phosphorus during skeletal development could result in seasonal differences in bone mineral content. The seasonal influence may be on calcium inclusion rather than elastin/collagen build up and this could explain the less marked differences found with QUS techniques compared with DEXA.

All infants were divided into six groups of increasing age and QUS variable mcSOS showed a statistical significant difference between age groups. After birth, we observed a decreasing trend of this value up to 240 days, then a peak around 270–300 days of life, a short deflection and a slow increase in the next months. Previous reports have shown that bone SOS particularly decreases in the early postnatal life of premature infants (Rubinacci et al., 2003). However, Liao et al. (2005) also observed this trend in full-term infants. Then Ritschl et al. (2005) found a reduction of mcSOS values up to 6 months of life and a subsequent increase with the achievement of birth values at 18 months of life. Our findings were in accordance with these previous observations. The reasons for this are not so clear; it might be a physiological phenomenon that requires further study, however, different hypotheses have been formulated. mcSOS reflects bone strength and it’s dependent on several bone parameters, including cortical thickness, density, microstructure, and elasticity, and it’s also influenced by soft tissue thickness. In the first months of life, the cortical thickness and density slightly decrease, as observed in experimental studies (Land & Schoenau, 2008). This observation might explain the decreasing trend in mcSOS values observed after birth in term infants. Indeed, it has been hypothesized that the reduction in postnatal values of mcSOS might be ascribed to BMI changes (Ritschl et al., 2005).

We found negative correlations between age and mcSOS and mcBTT. Similarly, in recent studies, the authors found a significant inverse correlation of QUS with postnatal age (Rubinacci et al., 2003; Tomlinson et al., 2006; Mercy et al., 2007; Ahmad et al., 2010). This was attributed to mineral deficiency and lack of mechanical stimulation, compared with the intrauterine situation. Also Rack et al. (2012) found a decline of QUS during the first weeks of life, which might reflect the negative correlation in other studies. The duration of this decline correlated with the maturity of the neonates, with a more prolonged decrease in the most premature infants.

In our study, QUS variables, mcSOS and mcBTT were related to anthropometric parameters, length and weight, reflecting bone size. These results agree with previous studies in both adults and children, suggesting that QUS values could be more influenced by bone size than thickness (Baroncelli et al., 2006). We found a statistical significant difference between weight groups only for mcBTT parameter. In the total sample, we observed negative correlations between mcSOS, length and weight, and between mcBTT and length. However in a multiple linear regression model of QUS parameters and age, weight, length and BMI at enrolment, we found a relation only between age and mcSOS and mcBTT.

According to literature data (Ritschl et al., 2005), a positive correlation between mcSOS e mcBTT has been observed. The large dispersion of the correlation can be due to the influence of surrounding soft tissues that differs mcSOS and mcBTT and uncertain ratio between the bone and soft tissues along the ultrasonic path.

We performed QUS assessment also in a small group of patients with rickets and we observed values of mcSOS and mcBTT lower than the 10th percentile of healthy infants’ group, but this observation needs further evaluation above a larger infants group and it’s only a suggestion for new specific studies. Then QUS technique could be useful to detect the presence of deficits in bone mineral status. In conclusion, the evaluation of bone health with QUS technique has multiple advantages, particularly in pediatric clinical practice. Furthermore, it could be a good tool to determine the role of different factors on programming of bone health.
